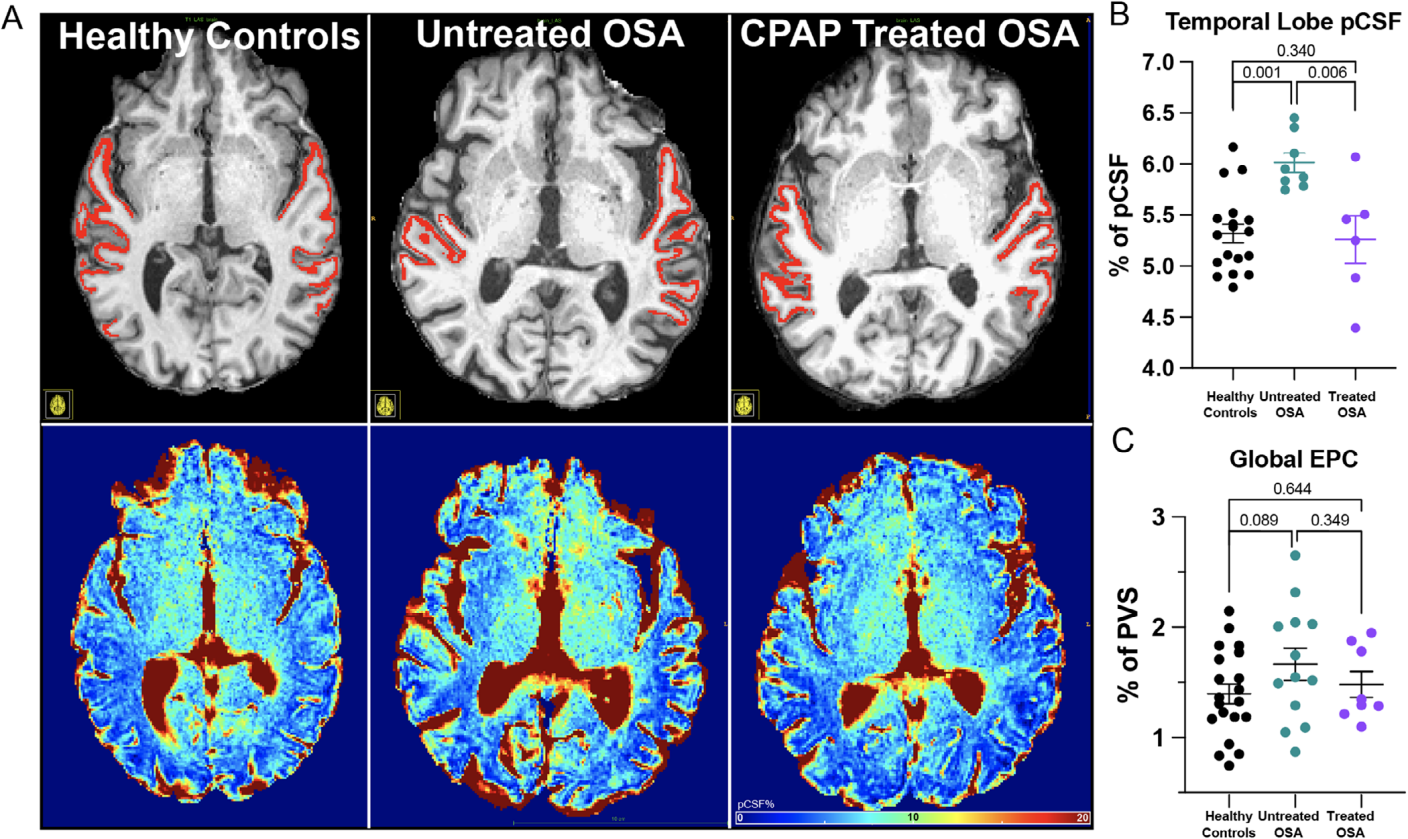# Increased glymphatic fluid volume in untreated obstructive sleep apnea

**DOI:** 10.1002/alz70856_104292

**Published:** 2025-12-25

**Authors:** Samantha A Keil, Liangdong Zhou, Xiuyuan Hugh Wang, Thanh D Nguyen, Gloria Chiang, Yi Li, Tracy A Butler

**Affiliations:** ^1^ Weill Cornell Medicine, New York, NY, USA; ^2^ Weill Cornell Medicine, New York City, NY, USA

## Abstract

**Background:**

Obstructive sleep apnea (OSA) affects an estimated 936 million adults globally. When left untreated, OSA increases risk of comorbid cardiovascular, metabolic, and neurodegenerative diseases including Alzheimer's. Continuous positive airway pressure (CPAP) remains the gold standard of treatment, ameliorating sleep apnea, reducing comorbidities, and improving brain health. However, difficulties with CPAP use results in 30‐80% non‐compliance. Identifying CPAP's therapeutic mechanisms may help develop alternative interventions for the millions who cannot benefit from CPAP.

Recent studies suggest OSA impacts brain fluid clearance, including the glymphatic system. The glymphatic system is a brain‐wide network of perivascular spaces (PVS) which facilitate clearance of metabolic waste during sleep. Clinical evaluation of glymphatic system PVS has proved challenging. MRI‐based enhanced PVS contrast (EPC) which defines enlarged white matter PVS, and novel whole brain parenchymal cerebrospinal fluid (pCSF) mapping which delineates CSF water fractions in PVS including pCSF offer potential solutions.

Here, we evaluate EPC and pCSF derived glymphatic fluid burden in CPAP‐treated and untreated OSA subjects and healthy age‐matched controls (HC).

**Methods:**

Cross‐sectional analysis (*n* = 32) involved HC, CPAP‐treated OSA, and untreated OSA groups. Neuroimaging included 3T MRI acquired T1W, multi‐echo FAST‐T2 for pCSF, and T2W sequences. Image processing was performed with FreeSurfer for ROI parcellation and an in‐house MATLAB code for pCSF mapping.

**Results:**

Across multiple regions, untreated participants exhibit the highest pCSF burden, while CPAP treatment appears to align closely with HC (Figure 1A‐B). In the temporal lobe, untreated OSA participants exhibited 6.01% pCSF which is significantly higher (*p* <0.05) than CPAP‐treated (5.26%) and HC (5.32%). Comparatively, there was no significant difference in EPC across all groups (Figure 1C 1.4‐1.7%). No difference in cortical volumes was observed across groups, suggesting glymphatic fluid findings using pCSF are not related to atrophy.

**Conclusion:**

These findings underscore the impact of OSA on the brain's glymphatic system, highlighting glymphatic dysfunction as a potential contributor to the downstream effect of untreated OSA. Comparative findings of EPC and pCSF suggest that pCSF may be a more sensitive approach to evaluating sublet changes in glymphatic fluid burden. Further research is necessary to substantiate these findings in a longitudinal within‐subject cohort before and after CPAP treatment.